# Persistent left superior vena cava mistaken for nodal metastasis: a case report

**DOI:** 10.1186/1752-1947-4-174

**Published:** 2010-06-09

**Authors:** Vasilios Tzilas, Antonios Bastas, Aspasia Koti, Dimitra Papandrinopoulou, Georgios Tsoukalas

**Affiliations:** 1 4th Respiratory Medicine Department, Athens Chest Disease Hospital Sotiria, Greece

## Abstract

**Introduction:**

Evaluation of the mediastinum is crucial for patients with lung cancer. Mediastinal lymph node metastases play a dramatic role in the process of staging. Physicians should be aware of the potential pitfalls regarding mediastinal evaluation. This case report provides an example.

**Case presentation:**

We report the case of a 57-year-old Caucasian man who presented with a four-month history of non-productive cough. He was diagnosed with non-small cell lung cancer. Initially, it was thought to be inoperable due to the presence of a para-aortic lymph node. A more careful examination of the mediastinum revealed that the "lymph node" was in fact a persistent left superior vena cava.

**Conclusions:**

This study highlights the difficulties in mediastinal staging, especially when intravenous contrast is not used. The recognition of this vascular malformation dramatically changed the therapeutic decisions, giving our patient the opportunity of surgical resection. To the best of our knowledge, such correlation has not been described in English literature.

## Introduction

Persistent left superior vena cava (PLSVC) is a rare vascular abnormality. It is, however, the most frequent abnormality of the mediastinal veins. The prevalence is estimated to be 0.3% in the general population. It is higher (up to 4.5%) in cases of congenital heart disease [1,2].

The key point for diagnosis is the identification of the course of the aberrant vessel. It begins from the left branchiocephalic vein (at the junction of the left subclavian and internal jugular veins) which is usually hypoplastic (65%). In 10 to 18% of cases there is absence of the (right) superior vena cava. PLSVC passes lateral to the aortic arch, anterior to the left hilum, crosses posterior to the posterior wall of the left atrium. It drains to the right atrium (90%) or, rarely, to the left atrium (10%). The latter case is frequently associated with atrial septal defects (ASD) and is a cause of shunt, usually of no clinical significance [3-5].

## Case presentation

**Figure 1:**
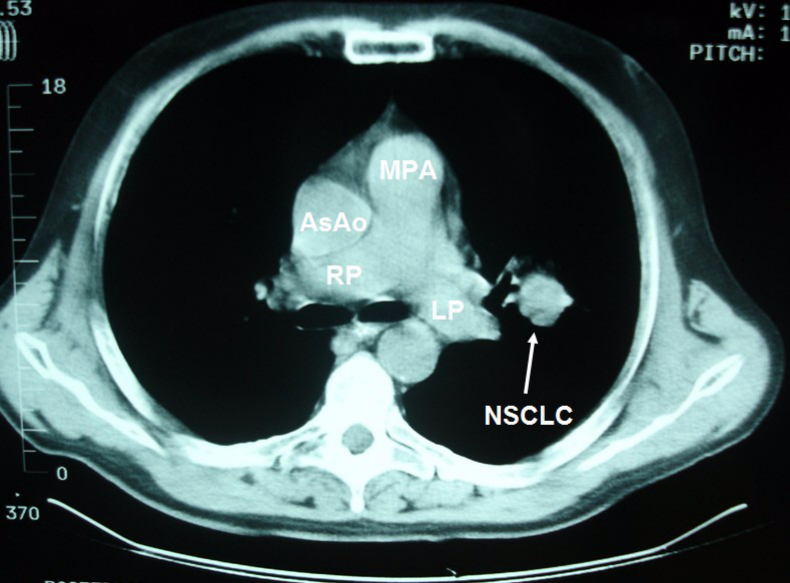
**Non small cell lung cancer in the left parahilar area**.

**Figure 2:**
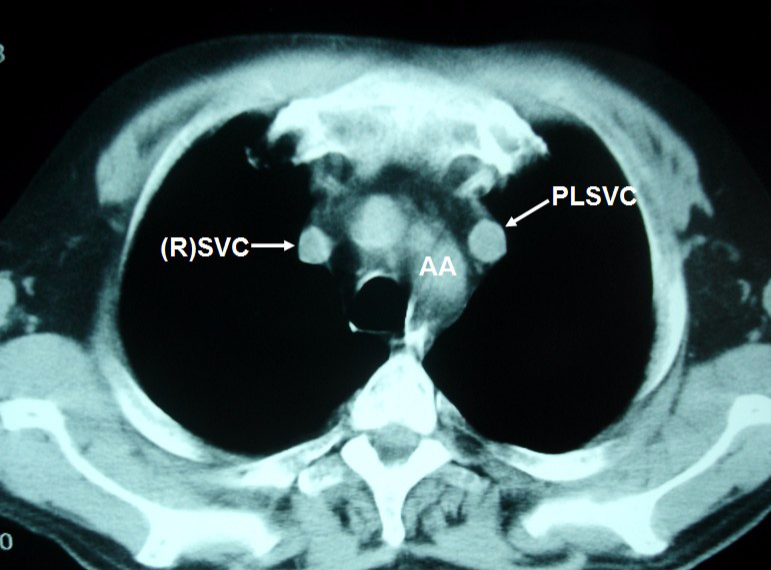
**PLSVC is seen as a nodule with anatomic correlation to the left branchiocephalic vein**.

**Figure 3:**
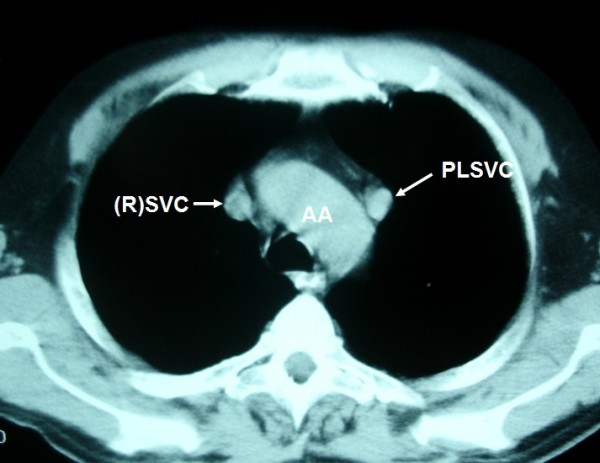
**PLSVC is seen as a nodular opacity lateral to the aortic arch in continuous levels**.

**Figure 4:**
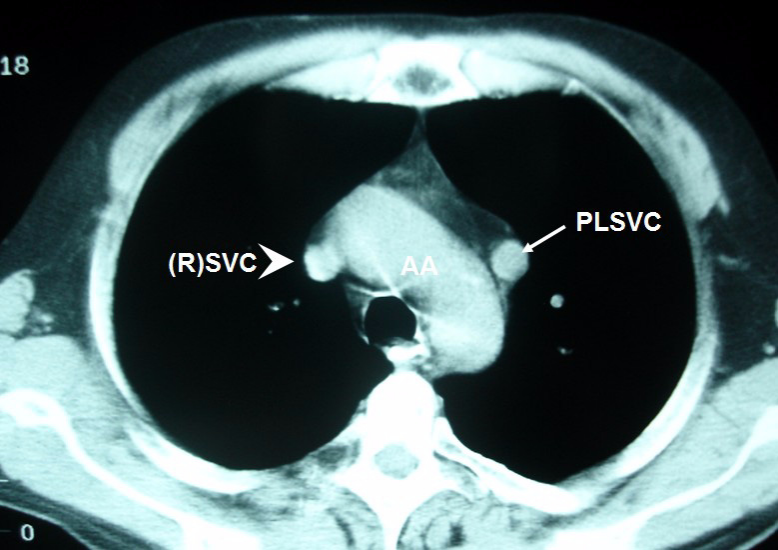
**In patients with PLSVC the "normal" (R)SVC (arrowhead) is present in 80 to 90%**.

**Figure 5:**
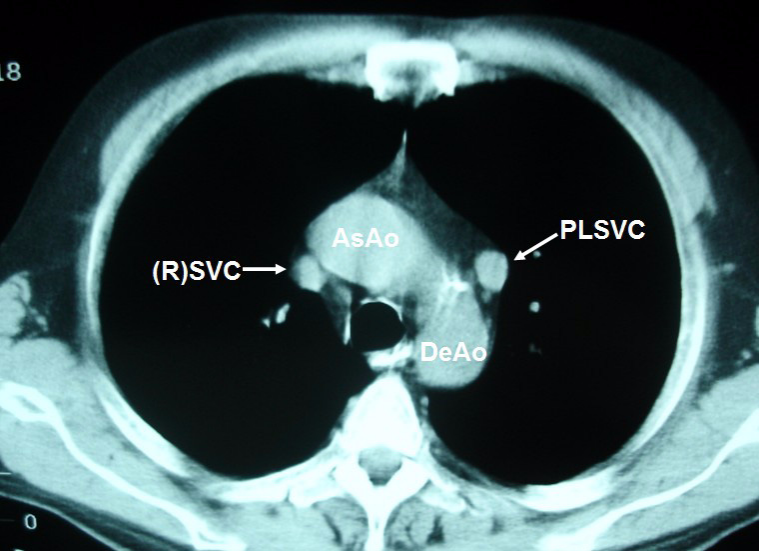
**Note the relatively small size of the (R)SVC**.

**Figure 6:**
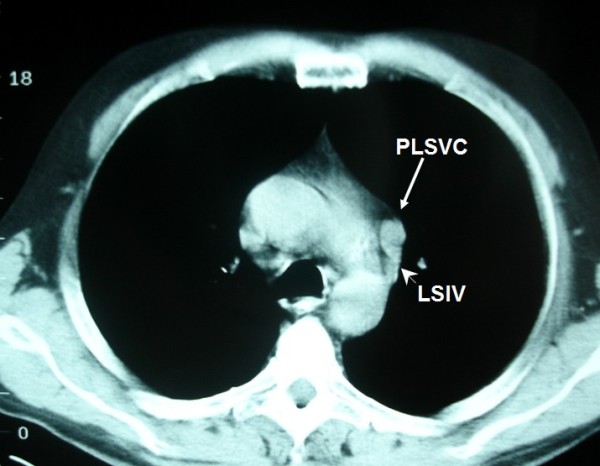
**LSIV is seen (arrowhead) emptying into the PLSVC (arrow) (hemiazygous arch)**. * AA: aortic arch; AsAo: ascending aorta; DeAo: descending aorta; LP: left pulmonary artery; LSIV: left superior intercostal vein; MPA: main pulmonary artery; NSCLC: non small cell cancer; PLSVC: persistent left superior vena cava; RP: right pulmonary artery; (R)SVC: (right) superior vena cava.

A 57-year-old Caucasian man presented to our clinic with a four-month history of chronic cough. He was a heavy smoker with a history of 80 packs per year. A chest X-ray revealed a nodule in the left upper lobe (LUL). A computed tomography (CT) scan of the thorax confirmed the presence of a round nodule in the LUL with smooth margins (Figure 1). It also revealed a "nodule" in the mediastinum, which was initially thought to represent N_2 _mediastinal lymph node (station 6-para-aortic, Figures 2, 3, 4, 5, 6).

During bronchoscopy there were no abnormal findings. Cytological evaluation of the obtained washings was negative for the presence of neoplastic cells. Finally, diagnosis was established with transthoracic fine needle biopsy which showed non small cell lung cancer (NSCLC). Initially, our patient was staged as IIIA, because of N2 disease. Thus, he was considered as a candidate for chemotherapy. However, a more detailed examination of the mediastinum revealed that the "nodule" was present in continuous levels. Therefore, it had an elongated shape. An elongated shape is characteristic of a tubular structure (e.g. a vessel) and is not seen in lymph nodes. Also, an anatomic correlation with the left branchiocephalic vein was identified. Finally, it is of great interest the absence of the azygous arch. There is, however, a prominent left superior intercostal vein (LSIV) which serves the same function creating a "hemiazygous arch" (Figure 6). Lymph nodes do not have branches. Hence, this finding is also compatible with the vascular nature of the "nodule".

Based on the above mentioned anatomic characteristics the diagnosis of PLSVC was established. The lack of intravenous contrast was a great disadvantage and resulted in the initial false staging.

A lobectomy (LUL) was performed. Histological examination of sampled lymph nodes during surgery was negative for malignancy. Our patient is free of disease at follow-up after two years.

## Discussion

Physicians should bear in mind that every node in the mediastinum is not a lymph node. The interpretation of CT scans should be made with extreme caution especially if intravenous contrast is not used. Each node should be examined in continuous levels. An elongated shape favors the possibility of a vascular structure. Possible anatomic relation to vessels should be sought as it will establish the diagnosis. The use of intravenous contrast is of utmost importance regarding mediastinal staging.

Nevertheless, sometimes intravenous contrast is not administrated (e.g. renal failure, allergies or even negligence). In such cases thorough knowledge of mediastinal anatomy (including normal variations) is essential in order to avoid mistakes.

## Conclusions

We presented a case of a 57-year-old man with an operable (as was proved surgically) NSCLC of the LUL. This case demonstrates the difficulties in mediastinal staging especially when intravenous contrast is not used. The patient had a congenital vascular abnormality. Diagnosing the left superior vena cava as a lymph node (lymph node station 6-para-aortic) would result in a false staging (IIIA, presence of N_2 _lymph node). The recognition of this vascular malformation changed dramatically the stage of the disease and therefore the therapeutic decisions and outcome of our patient.

## Abbreviations

CT: computed tomography; LSIV: left superior intercostal vein; NSCLC: non small cell cancer; PLSVC: persistent left superior vena cava.

## Competing interests

The authors declare that they have no competing interests.

## Authors' contributions

Each author participated equally in the diagnosis. All authors read and approved the final manuscript.

## Consent

Written informed consent was obtained from the patient for publication of this case report and any accompanying images. A copy of the written consent is available for review by the Editor-in-Chief of this journal.
